# IgG reactivity to different desmoglein-3 ectodomains in pemphigus vulgaris: novel panels for assessing disease severity

**DOI:** 10.3389/fimmu.2024.1469937

**Published:** 2024-10-02

**Authors:** Soheil Tavakolpour, Zahra Noormohammadi, Maryam Daneshpazhooh, Alireza Gholami, Hamidreza Mahmoudi

**Affiliations:** ^1^ Department of Biology, Science and Research Branch, Islamic Azad University, Tehran, Iran; ^2^ Autoimmune Bullous Diseases Research Center, Razi Hospital, Tehran University of Medical Sciences, Tehran, Iran; ^3^ Virology Department, Pasteur Institute of Iran, Tehran, Iran

**Keywords:** pemphigus vulgaris, desmoglein-3, IgG autoantibodies, ELISA, disease severity, ectodomains, PDAI, autoimmune

## Abstract

**Introduction:**

Pemphigus vulgaris (PV) is an autoimmune disease characterized by IgG autoantibodies targeting desmoglein-3 (Dsg3), leading to blistering of mucous membranes and skin. Although commercial ELISA kits effectively diagnose PV, correlation with clinical phenotype remains unclear. This study assesses multiple panels for monitoring disease severity and activity by profiling IgG autoantibodies against Dsg3’s various extracellular ectodomains.

**Method:**

We designed and expressed different extracellular domains of Dsg3 in HEK293T cell line and developed 15 different ELISA panels, each using a single or multi ectodomains encompassing the entire extracellular region of Dsg3 to detect specific autoantibodies against the particular part of Dsg3.

**Results:**

To validate our approach, we compared our ELISA panel for the full Dsg3 (EC1-5) against a commercial kit using 154 random serum samples from PV patients, demonstrating a strong correlation. For evaluation of IgG autoantibody profiles in our panels, 59 PV patients were included, along with 11 bullous pemphigoid patients, and 49 healthy controls. For all the included subjects, 15 predefined ELISA panels were tested. The IgG autoantibodies against EC1 were detected in 86% of patients with a positive full Dsg3 ectodomain (EC1-5) ELISA, with 26% against EC2, 14% for EC3, 29% for EC4, and 23% for EC5. Among the panels with multiple Dsg3 ectodomains, EC1-3 and EC1-4 were representative of the entire Dsg3 ectodomain in terms of ELISA positivity across all included patients. A significant correlation (P<0.05) was observed between ELISA optical density (OD) and Pemphigus Disease Area Index (PDAI) scores in five panels, EC1, EC2-3, EC2-5, and EC3-4 in addition to the full ectodomain. It suggests an association with disease severity. Interestingly, while the ELISA panel for the entire Dsg3 extracellular ectodomains did not differentiate disease phases, in three of our panels, including EC1, EC3-5, and EC2-5, ANOVA analysis showed a statistically significant difference between the groups of patients in remission, partial remission or persistent lesions, and those with active disease (new cases or relapse). Among these three panels, EC1 was the only one that showed a significant difference in the multiple comparisons analysis; patients in the active phase had higher levels of autoantibodies than those in ‘partial remission or persistent lesions’ and ‘complete remission’ groups.

**Conclusion:**

The level of autoantibodies against EC1 was not only correlated with the full ectodomain but also associated with higher disease severity and active disease phase. This study indicates that a detailed autoantibody profile against Dsg3 ectodomains could serve as a marker for PV severity and activity which may potentially enhance early treatment initiation.

## Introduction

Pemphigus is a rare group of autoimmune diseases that cause painful and flaccid blisters on the skin and mucous membranes (1). There are two major subtypes of pemphigus: pemphigus vulgaris (PV) and pemphigus foliaceus (PF). In PV, autoantibodies target desmoglein (Dsg) 3 and, in many cases, Dsg1 as well. These proteins are critical for maintaining the attachment of keratinocytes in the skin, as well as epithelial cells in mucosal layers, ensuring the integrity of both cutaneous and mucosal tissues (2). The autoimmune attack on these essential desmosomal proteins disrupts cell-to-cell adhesion between keratinocytes and epithelial cells, resulting in the loss of cohesion and breakdown of both the epidermal and mucosal layers, a process known as acantholysis. This process results in the formation of intraepidermal blisters that are characteristic of the disease. Since Dsg3 is more concentrated in the mucous membranes, particularly in the deeper layers, the presence of autoantibodies against Dsg3 typically leads to deep blisters in multiple mucosal regions, including the mouth, throat, and genitals (3, 4). Conversely, autoantibodies against Dsg1 tend to cause more superficial blisters that are often seen on the scalp, face, chest, and back (5).

Dsg3 is a crucial member of the desmosomal cadherin protein family and is essential for maintaining the structural integrity of epithelial tissues. This transmembrane glycoprotein, is predominantly expressed in mucous membrane epithelial cells such as those lining the oral cavity, esophagus, and genital mucosa. Dsg3 comprises multiple cadherin repeats in its extracellular domain, from EC1 to EC5 (6). These ectodomains are vital for calcium-dependent homophilic interactions, which promote strong cell-cell adhesion between adjacent molecules on neighboring cells. The intracellular domain of Dsg3 is also important, as it interacts with desmosomal plaque proteins, including plakoglobin and desmoplakin, and can trigger a signaling cascade following the binding of autoantibodies to the extracellular part of Dsg3 (7).

For the diagnosis of PV, the gold standard method is direct immunofluorescence (DIF) microscopy, which detects the deposition of IgG in the epidermis/epithelium of affected areas (8). However, this method is invasive. To utilize serum for diagnosis, monkey or guinea pig esophagus has been used as sensitive substrates for indirect immunofluorescence (IIF) microscopy, which detects circulating autoantibodies in the majority of pemphigus patients. Enzyme-linked immunosorbent assay (ELISA) is another technique that can be employed for the diagnosis of PV patients. One of the advantages of ELISA is that it is less dependent on the interpreter and can be quantified using controls with a known level of antibodies. Moreover, it is a very sensitive method and could show correlation with the disease severity in some patients (9).

The use of ELISA for diagnosing PV began in 1995 when the bacterial recombinant Dsg3 covering the EC1-EC2 part of Dsg3 was utilized (10). However, due to the expression source, some conformational epitopes may not be represented in the bacterial fusion proteins. Additionally, not encompassing the entire extracellular domain of Dsg3 could result in overlooking autoantibodies against other ectodomains. In a subsequent study by the same group, the entire extracellular domain of Dsg3 was used to infect “High Five” insect cells (11). In the latest generation of ELISA for PV patients, recombinant Dsg3 is expressed in a human cell line (HEK293) to ensure post-translational processing similar to the protein expressed on human keratinocytes (12). As previously mentioned, pathogenic antibodies in PV, which are presumed to interfere with cell-cell adhesion, can only bind to the mature form of the Dsg3 protein (13). Interestingly, HEK293 cells express the mature Dsg isoforms, making them an excellent source for producing recombinant Dsg3 (12–14). Currently, ELISA is a well-established method for the diagnosis of PV. Although the detection of anti-Dsg3 antibodies is sensitive and specific enough for the diagnosis of PV, there is some debate regarding the correlation between autoantibody levels and disease severity (15–19).

In the present study, we aimed to express different single and fused ectodomains in the mammalian cell line (HEK293) to evaluate the autoantibody profile in PV patients against different ectodomains of the Dsg3 and to investigate the accuracy of disease diagnosis as well as any potential correlation between this profile and disease characteristics.

## Methods

### Patients and serum samples

Serum samples from 59 patients with PV were obtained in the autoimmune bullous diseases clinic at Tehran University of Medical Sciences. All patients were clinically and histopathologically diagnosed, and each had a positive titer for anti-Dsg3 antibodies, as determined by ELISA at the time of diagnosis, using commercially available ELISA kit (Euroimmun, Luebeck, Germany). A physician completed a questionnaire for each patient to record disease characteristics and calculate the Pemphigus Disease Area Index (PDAI) score through physical examination, which was validated and confirmed by the second dermatologist (20). Disease phase, duration, and demographic data were also collected. Disease phases were defined as follows: 1) New case, 2) Relapse, 3) Partial remission, 4) Persistent lesion, and 5) Complete remission, according to the consensus (21). In addition to the PV patients, the study included 11 patients with bullous pemphigoid and 49 sex- and age-matched healthy controls. Blood samples were collected in tubes containing heparin, centrifuged at 600 g for 10 minutes, and then serum stored in a -80°C freezer for less than 6 months until ELISA testing was performed. In order to validate our ELISA results, 154 random frozen PV serum samples from Razi hospital biobank were included. All 154 samples were tested by ELISA using the FDA-approved kit (Euroimmun, Luebeck, Germany) as well as our constructed ELISA plate coated with the full extracellular ectodomains of Dsg3 (EC1-5). To enable comparison of results between FDA approved kit and the our developed ELISA kit, the same standard samples (for 2, 20, and 200 RU/mL, included in the commercial kit) were used to draw standard curves.

All included patients and healthy donors provided written consent to participate in the study. The study was conducted following the ethical standards outlined in the Declaration of Helsinki and followed the guidelines of the local ethics committees of Tehran University of Medical Sciences.

### Recombinant protein expression

A codon-optimized gene block for the coding region of the extracellular part of the human DSG3 gene was ordered as a double-stranded gene fragment from Integrated DNA Technologies (IDT). The fragment was directly cloned into a lentiviral expression plasmid (pLVX-EGFP-IRES-puro, addgene #128652) engineered to have gene of interest after signal sequence and include a His-tag at the C-terminus. Additionally, two restriction sites for *Eco*RI and *Bam*HI were introduced after the signal sequence and before the His-tag, respectively. This plasmid served as a backbone for further cloning and was used as a transfer plasmid. Specific primer pairs were designed to amplify different segments of the entire extracellular domain, each with a 15-20 bp overlap. After confirming the size of the fragments on 1% agarose gel, Gibson Assembly (22) method was used to clone the amplified fragments into the double-digested template vector. Following confirmation of the nucleotide sequence by Sanger sequencing for each construct, the final plasmid was used as a transfer vector.

Given the importance of correct folding and post-translational modifications for Dsg3, all peptides were expressed in mammalian cell line. To this end, HEK293T cells were co-transfected with the lentiviral transfer plasmid expressing particular ectodomain(s) of Dsg3, the pCMV-VSV-G envelope plasmid (Addgene #8454), and the pCMV-Pax2 packaging plasmid (Addgene #36052) using polyethylenimine (PEI, Sigma-Aldrich) according to the manufacturer’s instructions. The supernatant containing the lentivirus was harvested at 48hours post-transfection and filtered through a 0.45-micron PES membrane. It was then concentrated at 30,000x g for 2 hours at 4°C and directly used to transduce HEK293T cells, with transduction efficiency enhanced by Polybrene (5 µg/mL). The protein-producing cells were cultured and expanded in DMEM supplemented with 10% fetal bovine serum (FBS), 1% Penicillin/Streptomycin, 1 mM sodium pyruvate, and non-essential amino acids in the 15 cm² culture plate. After selection with puromycin (2 μg/mL), the cells were cultured until they reached 90% confluence, at which point the medium was switched to serum-free conditions. The culture media were collected every 3 days for 3 cycles, and the protein was purified from the harvested media using IMAC chromatography on Ni-NTA beads. For each protein, expression and specificity were first confirmed by western blot, followed by analysis of purity using 10% SDS-PAGE.

### Enzyme-linked immunosorbent assay

Sandwich ELISA was used to detect antibodies in the patients’ serum. ELISA plates were coated overnight at 4°C with a 5 µg/ml solution of purified Dsg3. After washing the plates three times with the washing buffer (PBS containing 0.1% Tween-20 and 0.8 mM calcium), plates were blocked with PBS + 0.8 mM calcium containing 4% BSA for 1 hour at room temperature. Serum samples, diluted 1:100 in PBS with 1% BSA and 0.8 mM calcium, were incubated on the plates for 2 hours at room temperature. The plates were then washed three times and incubated with horseradish peroxidase (HRP)-conjugated anti-human IgG antibody (Invitrogen, 1:2500) containing 0.8 mM calcium for 1 hour at room temperature. Following another three washes, TMB (3,3’,5,5’ tetramethylbenzidine) solution (Biolegend) was added and incubated for 10 minutes. The reaction was stopped by adding 2 N H2SO4, and absorbance was measured at 450 nm using a spectrophotometer. All samples were tested in duplicate, and the average values were reported. Samples with more than 5% variation between replicates were re-tested.

### Data analysis

Optical density (OD) was determined at 450 nm. The cut-off for positivity using negative control was optimized by receiver-operating characteristics (ROC). Quantification was performed to facilitate comparison between our ELISA kit and the reference commercial kit, using relative units (RU) calculated from the OD values with standard curves drawn from three standards of 2, 20, and 200 RU/mL provided by the commercial kit (Euroimmun, Luebeck, Germany).

The data was analyzed and presented using GraphPad Software, Inc, Version 9. A two-tailed unpaired Student’s t-test was used for statistical comparisons between two groups. In the case of parametric analyses, comparisons were made using a one-way analysis of variance (ANOVA) as well as multiple comparisons analysis for three groups or the t-test for two groups. A p<0.05 was considered statistically significant.

## Results

### Patients’ and healthy controls’ characteristics

We tested the serum of 59 PV patients, in addition to 11 BP patients and 49 healthy individuals as negative controls. The mean ages of the PV patients, BP patients, and healthy controls were 50.7 ± 11.7, 62.7 ± 12.7, and 46.4 ± 11.6 years, respectively. **Table 1** summarizes the patient characteristics data at the time of sample collection.

**Table 1:** Patients’ and healthy controls’ characteristics.

		PV	BP	HC
Number		59	11	49
Age (mean ± SD)		50.7 ± 11.7	62.7 ± 12.7	46.4 ± 11.6
Gender				
	Male	29	5	21
	Female	30	6	28
Disease duration (Year, Mean ± SD)		4.4 ± 4.1	2.3 ± 2.2	n/a
Disease phase				
	New case	5		
	Relapse	18		
	Persistent lesions	7		
	Partial remission	14		
	Complete remission	6		
	Undetermined	9		
PDAI (median ± IQR)		4 ± 6		
Clinical phenotype				
	Cutaneous	4		
	Mucosal	10		
	Mucocutaneous	36		
	Undetermined	9		
Medication				
	Prednisolone	51		
	Rituximab*	23		
	Off-therapy	8		

PV, pemphigus vulgaris; BP, bullous pemphigoid; HC, healthy controls; PDAI, pemphigus disease area index.

*Within the 6 months before taking serum sample. n/a, not applicable.

### Validation of Dsg3 protein functionality

**Figure 1:**
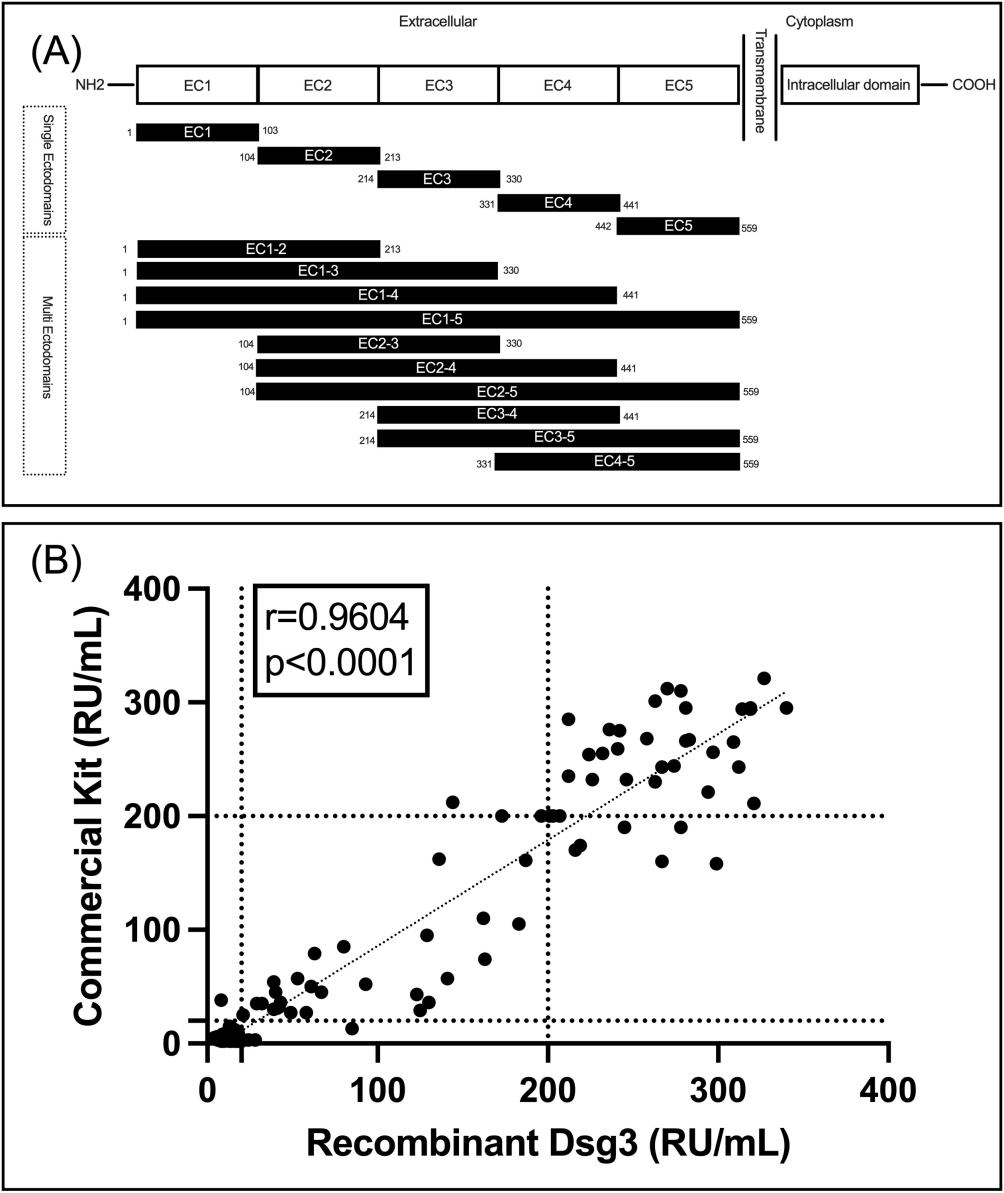
**(A)** schematic of designed and expressed peptides; **(B)** Correlation between the commercial kit and our Dsg3(EC1-5)-coated ELISA plate for detection of IgG autoantibodies.

All 15 peptides of Dsg3, encompassing single and multiple ectodomains, were successfully expressed and confirmed by SDS-PAGE and Western blot for assessment of purity and specificity, respectively (**Supplementary Figure S1**). **Figure 1A** shows the schematic of the peptide fragments that were expressed. After confirming protein expression and purity, we assessed the functionality, sensitivity, and specificity of our expressed Dsg3 EC1-5 in comparison to a commercially available FDA-approved kit. It is used for the detection of IgG autoantibodies against the extracellular part of human Dsg3. To enable comparison of results, the same standard samples (for 2, 20, and 200 RU/mL, included in the commercial kit) were used to draw standard curves. To validate our ELISA, 154 frozen serum samples were included from biobank, and ELISA tests were performed using both the commercial ELISA plate (as the reference) and the ELISA plate coated with recombinant Dsg3 expressed in the HEK293T cell line. Interestingly, there was a significant correlation between results for two ELISA tests (r=0.9604, P<0.0001). For the commercial kit, among the 154 samples, 85 were below 20 RU (considered negative, with a mean of 4.7 RU) and 69 were equal to or higher than 20 RU (considered positive, with a mean of 168.7 RU). Regarding the results for the ELISA test with our expressed Dsg3, 76 samples were below 20 RU (mean 13.1 RU) and 78 were equal to or above 20 RU (mean of 172.1 RU). Out of the 85 samples deemed negative by the commercial kit, 8 showed positive results when using our Dsg3-coated ELISA plate. This suggests an accuracy of 90.5% for the detection of negative samples (with a false-positive rate of 9.5%). Regarding the positive results from the commercial kit, 68 out of 69 samples were also positive in our Dsg3-coated ELISA, indicating an accuracy of 98.6%. This suggests that our ELISA tests are reliable in detecting positive samples, with a false-negative rate of only 1%. **Figure 1B** details our findings in a scatter plot and visualizes the positive correlation between the two approaches for the detection of IgG reactive autoantibodies against Dsg3 (EC1-5).

### Cut-off for each reactivity against each ectodomain and immunoreactivity

The results of the ELISA for the detection of IgG autoantibodies against the various fragments of Dsg3 were reported and analyzed based on the OD at 450 nm. For Dsg3 EC1-5, the OD corresponding to 20 RU/mL was considered the cutoff (according to the commercial kit manual). Although no positive control was available for the other 14 peptides, we attempted to validate our data to check the sensitivity, specificity, and false-positive rate for each fragment using statistical analysis.

For the evaluation of sensitivity and specificity, it is important to consider that not all ectodomains are necessarily positive in patients with positive Dsg3 (EC1-5) results. However, patients with negative results are most likely negative for all ectodomains. Therefore, specificity could be considered as a crucial factor in demonstrating the rate of false positives using the calculated cutoffs.

Out of the 59 included PV patients, 35 were detected with positive IgG anti-Dsg3 (EC1-5), and 24 were negative, resulting in a positive rate of 59.3%. Considering only patients who are positive for at least one epitope (positive results for the whole extracellular part of Dsg3) and the calculated optimal cutoffs, we found that 86% of patients have autoreactive IgG antibodies against Dsg3 EC1, 26% against EC2, 14% for EC3, 29% for EC4, and 23% for EC5. However, for multi-ectodomains of Dsg3, 100% of the included patients were positive for EC1-3 and EC1-4 in our study.

**Figure 2:**
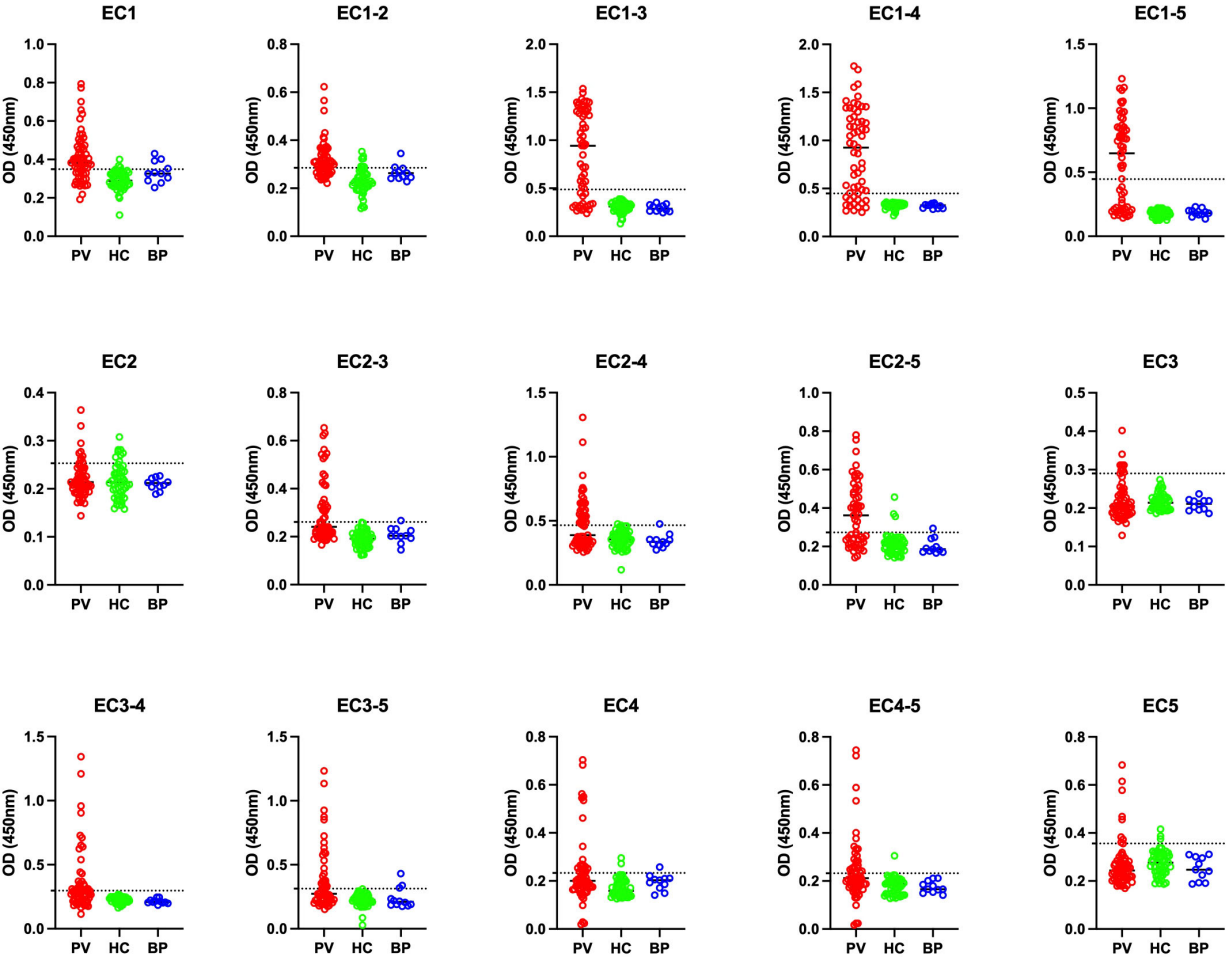
Comparison of optical density (OD) at 450 nm among pemphigus vulgaris (PV), bullous pemphigoid (BP), and healthy control (HC) groups across 15 different panels. PV, pemphigus vulgaris; BP, bullous pemphigoid; HC, healthy controls. The optimal cutoff is indicated by a horizontal dashed line for each panel.

Regarding the positivity of controls, EC1-2, EC2, and EC1 showed the least accuracy, with false-positive rates between 10% to 15%. However, the false-positive rate was 0% for EC1-3 and EC1-4. This suggests that these two fragments could be potential alternatives to Dsg3 EC1-5 in diagnostic perspectives (**Table 2**; **Figure 2**).

**Table 2:** calculated cut-offs, sensitivity, and specificity for each panel.

Group	Sensitivity	Specificity	Optimal Cutoff	Patients Positive (%)*	Patients Negative (%)*	Controls Positive (%)	Controls Negative (%)
**EC1**	0.857	0.883	0.349	30 (85.7%)	5 (14.3%)	7 (11.7%)	53 (88.3%)
**EC1-2**	0.828	0.85	0.285	29 (82.9%)	6 (17.1%)	9 (15.0%)	51 (85.0%)
**EC1-3**	1	1	0.497	35 (100%)	0 (0%)	0 (0%)	60 (100%)
**EC1-4**	1	1	0.448	35 (100%)	0 (0%)	0 (0%)	60 (100%)
**EC2**	0.257	0.867	0.253	9 (25.7%)	26 (74.3%)	8 (13.3%)	52 (86.7%)
**EC2-3**	0.657	0.983	0.261	23 (65.7%)	12 (34.3%)	1 (1.7%)	59 (98.3%)
**EC2-4**	0.771	0.967	0.465	27 (77.1%)	8 (22.9%)	2 (2.9%)	58 (97.1%)
**EC2-5**	0.943	0.933	0.273	33 (94.3%)	2 (5.7%)	4 (6.7%)	56 (93.3%)
**EC3**	0.143	0.983	0.29	5 (14.3%)	30 (85.7%)	1 (1.7%)	59 (98.3%)
**EC3-4**	0.571	0.967	0.297	20 (57.1%)	15 (42.9%)	2 (2.9%)	58 (97.1%)
**EC3-5**	0.657	0.967	0.313	23 (65.7%)	12 (34.3%)	2 (2.9%)	58 (97.1%)
**EC4**	0.286	0.917	0.233	10 (28.6%)	25 (71.4%)	5 (8.3%)	55 (91.7%)
**EC4-5**	0.429	0.95	0.232	15 (42.9%)	20 (57.1%)	3 (5.0%)	57 (95.0%)
**EC5**	0.229	0.95	0.356	8 (22.9%)	27 (77.1%)	3 (5.0%)	57 (95.0%)

*Only positive patients for anti-Dsg3(EC1-5) were included (n=35).

### Correlation between IgG reactivity profile and disease phenotype

**Figure 3:**
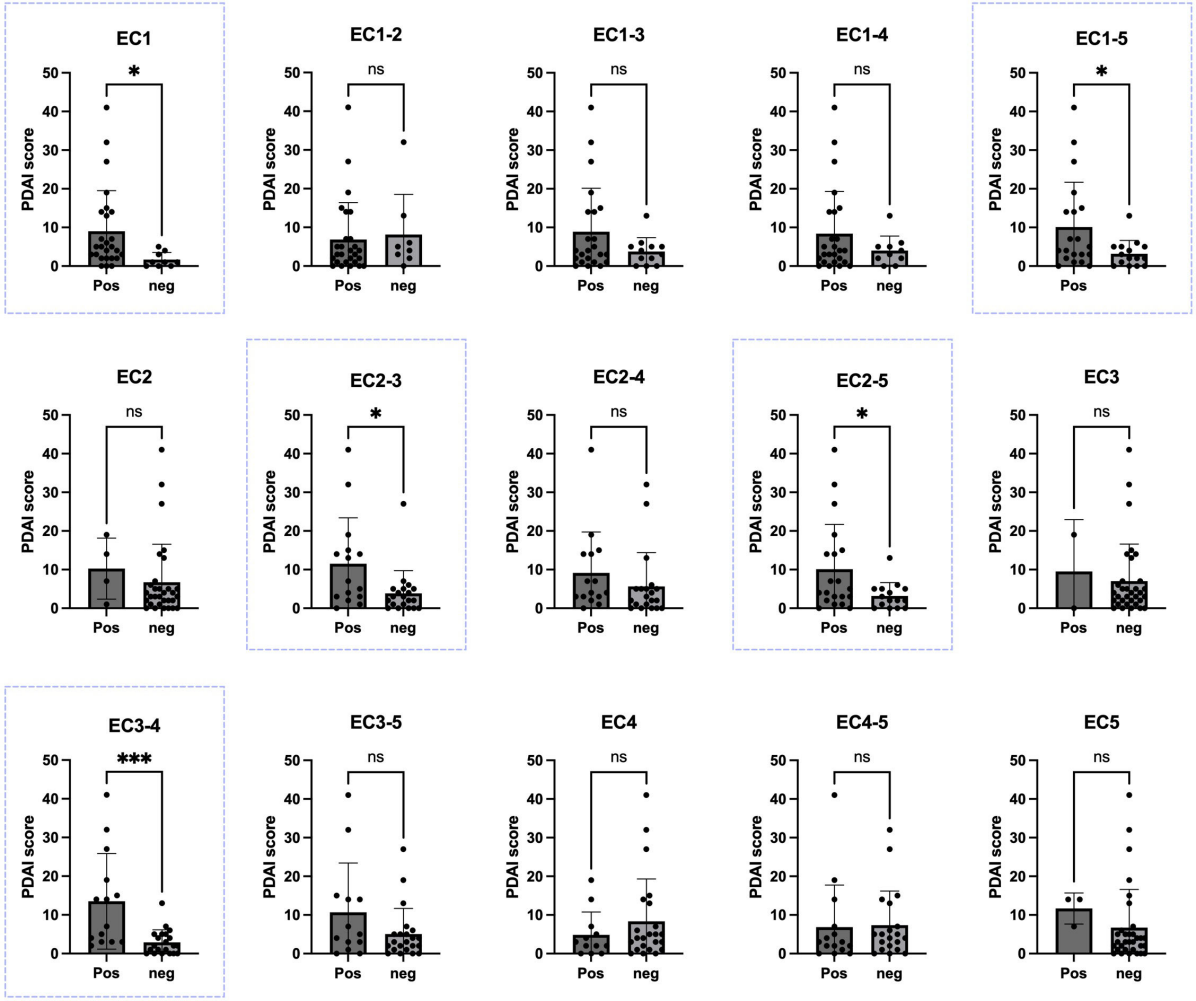
Correlation between Pemphigus Disease Area Index (PDAI) scores and the presence of autoantibodies against various extracellular portions of desmoglein-3 (Dsg3). In five panels of EC1, EC2-3, EC2-5, EC3-4, and EC1-5 (blue boxes). There was a significant difference in the PDAI scores between PV patients with positive ELISA results and those with negative ELISA results, based on each calculated cutoff for each panel. Significance levels are indicated as follows: ✱: P < 0.05, ✱✱✱: P < 0.001. Pos, positive; Neg, negative; PDAI, Pemphigus Disease Area Index. ns, not significant.

Disease severity was assessed using the PDAI scoring system through clinical examination of 35 patients (PDAI score was missing for 24 patients). Initially, for the evaluation in each panel, patients were categorized into two groups: those with an OD higher than the cutoff (considered positive) and those with an OD lower than the cutoff (considered negative). Subsequently, parameters related to disease severity were compared within each group. Regarding the PDAI score, analyses for linear correlation between the score and OD values, in addition to comparing the mean score between positive and negative groups, were performed. A significant positive correlation (P<0.05) was shown between the OD values and PDAI for EC1-5 (p-value: 0.0332), EC1 (p-value: 0.0453), EC2-3 (p-value: 0.0167), EC2-5 (p-value: 0.0332), and EC3-4 (p-value: 0.0006). These ectodomains were associated with disease severity score in our study (**Figure 3**).

**Figure 4:**
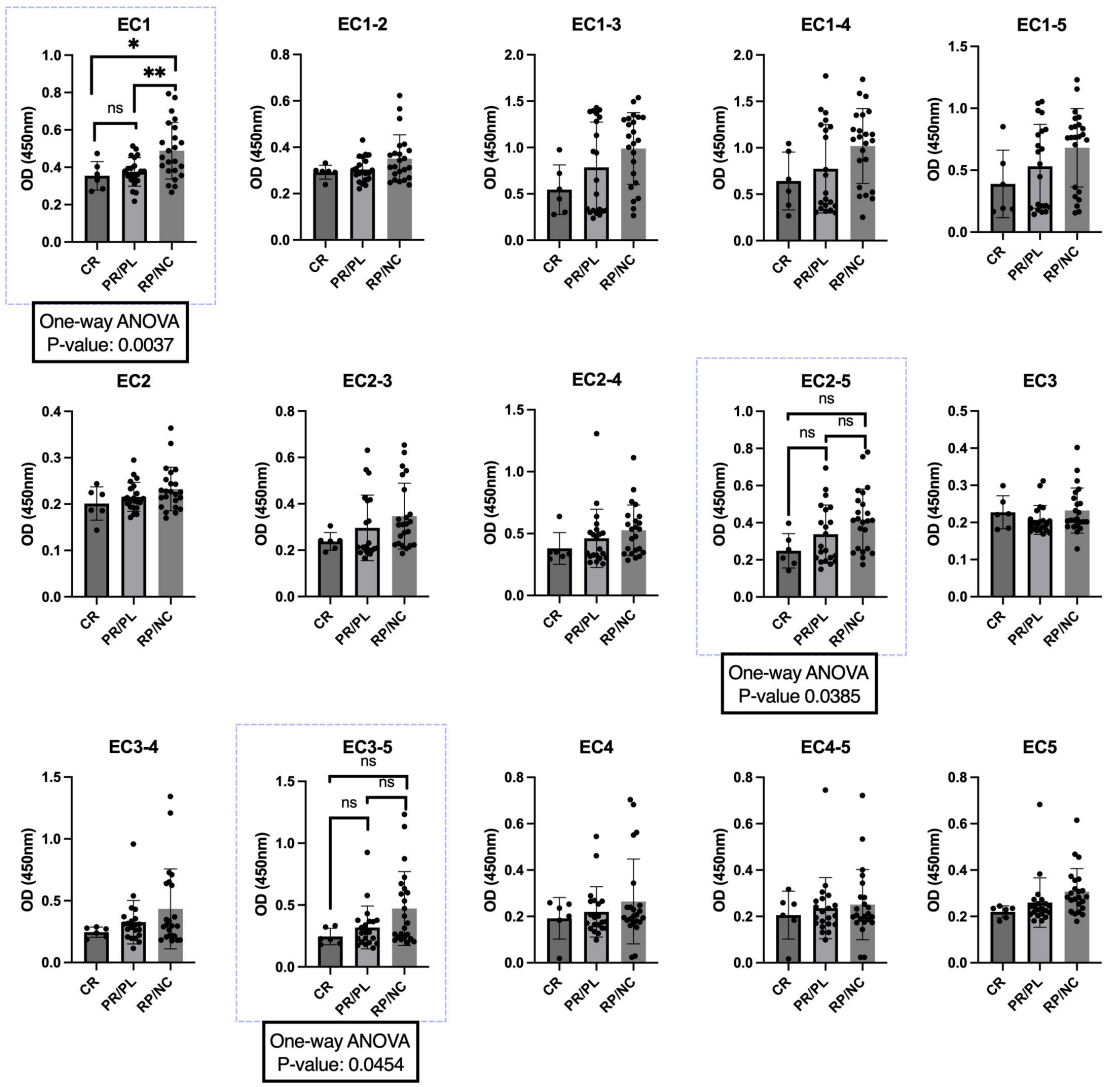
Association between disease phase and optical density (OD) at 450 nm for different extracellular portions of desmoglein-3 (Dsg3). Using a one-way ANOVA test, there was a significant difference between the groups in the EC1, EC2-5, and EC3-5 panels (blue boxes). P-values are shown in the box, with P-values for multiple comparisons indicated on top of each pair of columns. Significance levels are denoted as follows: ✱: P < 0.05, ✱✱: P < 0.01. CR, complete remission; PR, partial remission; PL, persistent lesions; RP, relapse; NC, new case. ns, not significant.

We also analyzed OD values in each group of patients with different disease phases for each ectodomain. Patients were categorized into three groups: 1) complete remission, 2) partial remission or persistent lesions, and 3) new cases or relapse. Although no difference was found between the groups in the Dsg3 (EC1-5) panel, one-way ANOVA analysis showed a significant difference among the disease phase categories for Dsg3 (EC1), Dsg3 (EC3-5), and Dsg3 (EC2-5) panels (P-values: 0.0037, 0.0454, and 0.0385, respectively). In the EC1 panel, multiple comparisons analysis also showed significant differences between the groups; specifically, patients in the “new cases or relapse” group had the higher OD than those in the “partial remission or persistent lesions” (P-value: 0.0069) and “complete remission” (P-value: 0.0426) groups (**Figure 4**). However, in EC2-5, and EC3-5, despite the significant difference among the groups, we did not detect significant differences in multiple comparisons analysis of groups.

No significant differences were observed between the results in each panel and disease duration, clinical presentation of lesions, or demographic data. We also evaluated the potential correlation between the total number of positive ectodomains among those positive for Dsg3 EC1-5 (out of 14). No significant correlation was found with disease phase, PDAI, disease duration, or demographic data.

## Discussion

In the present study, we evaluated the IgG autoantibody profile in patients with PV against different ectodomains of the Dsg3. We expressed various ectodomains of Dsg3 separately, including single domains to assess antibodies that bind directly to their epitopes independently of neighboring ectodomains, as well as multi-ectodomains, which cover not only epitopes on a single ectodomain but also those that are dependent on neighboring ectodomains for binding to autoantibodies. According to our results, IgG autoantibodies that bind to epitopes on EC1 are present in 86% of patients with positive ELISA for the entire extracellular part of Dsg3, although this may be influenced by many factors, including disease phase and severity. Other single ectodomains were not as immunogenic compared to EC1. These findings are consistent with previously reported data that emphasized the high immunogenicity of the N-terminal (EC1) domain of Dsg3 in PV patients as compared to the other ectodomains (23, 24). This may be due to the crucial role of the N-terminal in mediating both homophilic and heterophilic interactions (6).

Two major mechanisms are suggested for the effect of antibodies on keratinocytes. The first is related to the physical hindrance of the interaction between Dsg3 and desmocollin (DSC). In this regard, binding to ectodomains closer to the N-terminus of Dsg3 might lead to more potent outcomes, since Dsg3 primarily interacts with DSC from the N-terminus side (25, 26). The second mechanism involves the induction of intracellular signaling pathways in keratinocytes, leading to the clustering and internalization of surface Dsg3 or the activation of signaling cascades that result in cell-cell detachment (27–29). Both physical hindrance and the induction of intracellular signaling pathways may depend on the targeted epitopes affected by autoantibodies. Our results indicate that the presence of autoantibodies against EC1 is associated with higher disease severity and a more active phase of the disease, which may be explained by the importance of this ectodomain in epithelial adhesion and/or the induction of intracellular signaling pathways toward more inflammatory conditions.

We designed panels with single and multi-ectodomains to cover the potential effects of neighboring ectodomains. It is plausible that having a folded protein containing multiple ectodomains can recruit different sets of autoreactive antibodies compared to having single ectodomains separately. Epitopes located between ectodomains or changes in the physical shape of the protein, which can make some epitopes more or less accessible, could be the reasons (6). Understanding the profile of autoantibodies against different ectodomains of Dsg3 in patients can be useful for predicting outcomes and could also be beneficial in novel targeted therapies, such as chimeric autoantibody receptor (CAAR) T cells, which include all the required ectodomains on the surface of killer cells (30). It is also useful as a marker for disease severity. Having a serological marker for disease can help clinicians initiate treatment earlier. Since the development of ELISA kits for the detection of autoantibodies, the question of whether an anti-Dsg3 profile can be representative of clinical presentation of disease has been challenging. Some studies suggest that higher IgG anti-Dsg3 antibodies are associated with higher disease severity (12, 31). However, other studies did not find anti-Dsg3 ELISA results as an appropriate marker correlated with disease phenotype/severity (32, 33).

In multiple studies, it was reported that circulating anti-Dsg3 IgG autoantibodies in considerable PV patients in clinical remission showed positive results for anti-Dsg3 antibodies using ELISA (18, 34). This discrepancy might be explained by an improper cutoff for Dsg3 or different technical settings (34). For example, it was shown that using inappropriate dilution could cause a failure to show a decline in anti-Dsg3 antibody levels despite mucosal clinical remission (15). Another reason could be the lag time between autoantibody secretion/clearance and the reflection of the clinical phenotype. The ability of the anti-Dsg3 ELISA to predict disease relapse in patients in clinical remission supports this hypothesis (35). Inability to detect only pathogenic autoantibodies against specific ectodomains/epitopes could be another explanation, since not all the autoantibodies are pathogenic (35). In fact, PV patients possess a polyclonal autoantibody mixture, indicating that both pathogenic and non-pathogenic antibodies coexist. Current commercial ELISA kits, as well as in our study, detect only total IgG in the serum of patients. However, regardless of the different light and heavy chains, each subtype has different pathogenicity. In PV, the pathogenic subtypes seem to be IgG1 and IgG4, with the former mediating tissue damage and the latter mainly mediating acantholysis and dominating the autoimmune response (36). The differences between IgG subclasses could be due to the unique profile of effector activities in each subtype, although heavy chains share significant sequence homology. Regardless of subclass, binding to the N-terminal of Dsg3 and in a calcium-dependent manner are considered indicators of high pathogenicity (37), although binding of autoantibodies to the other epitopes may induce additional and/or synergistic effects. However, recent findings have shown that targeting EC5 in a calcium-independent manner can also have pathogenic effects (38).

In this study, we demonstrated that the detection of IgG autoantibodies against the entire extracellular domain of Dsg3, specifically EC1, EC2-3, EC2-5, and EC3-4, correlated with the PDAI. Regarding disease phases, the IgG autoantibody level against the entire Dsg3 ectodomain (EC1-5), commonly used in commercial kits, showed no significant difference between patients with active disease and those in complete or partial remission. However, the panel for detecting autoantibodies against certain portions of the extracellular domain of Dsg3, including EC1, EC2-5, and EC3-5, revealed significant differences between groups with varying disease phases.

Previous studies have indicated that the pathogenicity of autoantibodies targeting the EC3, and EC4 regions is lower compared to those binding to EC1 and EC2. The importance of EC1 and EC2, could be because of their crucial roles in cis-adhesive interaction. Although autoantibodies against other single subdomains, including EC3, EC4 and EC5 are detectable in active disease, they poorly or do not induce acantholysis (39). It is worthy to note that in a single cell analysis of two PV patients, in addition to antibodies that bind to EC1 and EC2, several pathogenic EC4-specific antibodies were also identified (40).

Although the presence of autoantibodies targeting EC1 was associated with more severe disease and more active phases, we found the association between the detection of autoantibodies against the EC2-3, EC2-5, and EC3-4 regions with disease severity scores, as well as the detection of autoantibodies against the EC2-5 and EC3-5 peptides with disease phase, which was unexpected. This finding suggests that critical epitopes might be more exposed to antibodies due to the specific folding of peptides containing multiple ectodomains. However, our analysis of the panels’ results in multiple comparisons did not show significant correlations between the EC2-5 and EC3-5 panels and disease phases. Thus, further studies are required to assess the capacity of these panels to predict disease severity in PV patients.

Our observations in this study regarding different results when we use different ectodomains of Dsg3 could be used to design more sensitive ELISA tests for monitoring disease activity. The reason for the differences in results could be the presence of non-pathogenic autoantibodies that could bind to specific epitopes when the entire extracellular Dsg3 is used in ELISA. Additionally, antibodies against regions close to the cell membrane are not deeply involved in keratinocyte adhesion and might cause positive ELISA results with low or no clinical phenotype. For example, having antibodies only against EC5 can lead to the detection of autoantibodies via available commercial kits, but there is no significant association between these antibodies and disease severity or phenotype.

Some limitations in our study should be mentioned. Firstly, we only evaluated the presence of autoantibodies based on their binding to any epitope on either a single ectodomain or in the presence of some neighboring ectodomains, although they might have different affinities or capabilities to disrupt keratinocyte adhesion, which ELISA cannot detect. However, as discussed earlier, not all of these antibodies are pathogenic. There are multiple possible epitopes for antibodies within a single ectodomain, each potentially associated with different levels of pathogenicity. Approaches to distinguish between antibodies based on pathogenicity, such as adding EDTA to chelate calcium and excluding potentially non-pathogenic autoantibodies, have been suggested (41). Experiments such as dissociation tests using human keratinocyte cell lines (e.g., HaCaT), which were outside the scope of our study, could also be informative. Secondly, although all the expressed fragments were produced in mammalian cell lines to ensure correct folding and glycosylation, which is crucial since the majority of epitopes on Dsg3 are conformational, the folding of Dsg3 ectodomains in the native Dsg3 form might differ from the folding when one or more ectodomains are missing, which could affect the binding of autoantibodies. Moreover, lack of a positive control for each panel, the presence of false positives in some panels, and the small sample size for determining optimal cutoffs might affect the results. In this study, we have detected total IgG antibodies that that bind to each of expressed Dsg3 ectodomains. Thus, we were not able to distinguish between the subtypes. However, it has been reported that IgG1 and IgG4 are major involved pathogenic subtypes in PV patients. It could be speculated that subtype be associated with disease severity and identification of subtypes might increase the specificity of ELISA.

In conclusion, EC1 was found to be the most frequently targeted by autoantibodies in PV patients; autoantibodies against entire extracellular Dsg3 (EC1-5), EC1, EC2-3, EC2-5, and EC3-4 were associated with disease severity, and autoantibodies against EC1, EC3-5, and EC2-5 were associated with disease phase. Detection of autoantibodies against specific ectodomains of Dsg3 could lead to more accurate diagnosis of PV and has greater clinical relevance.

## Data Availability

The raw data supporting the conclusions of this article will be made available by the authors, without undue reservation.

## Supplementary material

Supplementary Figure 1 Showing the specificity and purity of the expressed ectomdomains by **(A)** Western blotting using HRP-labeled secondary antibody and **(B)** Coomassie blue staining of 10% SDS-PAGE, respectively. Note that each lane was run on separate gels under identical conditions. The images have been aligned and assembled for comparison purposes.
