# Supplementary figures and images for: IgG reactivity to different desmoglein-3 ectodomains in pemphigus vulgaris: novel panels for assessing disease severity
Source: Front Immunol. 2024 Oct 2;15:1469937. doi: 10.3389/fimmu.2024.1469937 (PMC11479927; doi:10.3389/fimmu.2024.1469937)

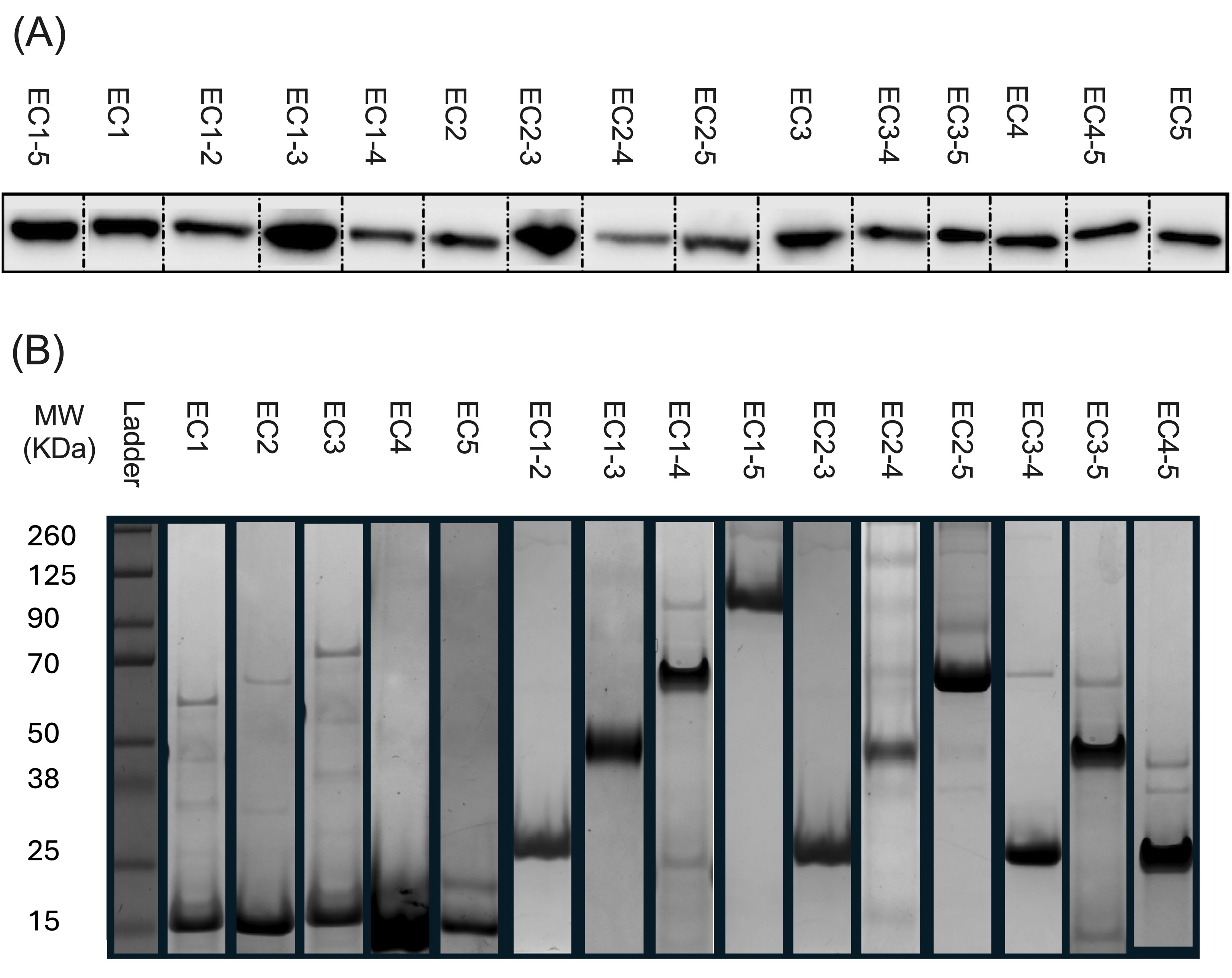

Supplement: Supplementary Figure 1 — Showing the specificity and purity of the expressed ectomdomains by (A) Western blotting using HRP-labeled secondary antibody and (B) Coomassie blue staining of 10% SDS-PAGE, respectively. Note that each lane was run on separate gels under identical conditions. The images have been aligned and assembled for comparison purposes. [file Image1.jpeg]
